# A Selective GSK3β Inhibitor, Tideglusib, Decreases Intermittent Access and Binge Ethanol Self‐Administration in C57BL/6J Mice

**DOI:** 10.1111/adb.70044

**Published:** 2025-05-19

**Authors:** Sam Gottlieb, Andrew van der Vaart, Annalise Hassan, Douglas Bledsoe, Alanna Morgan, Brennen O'Rourke, Walker D. Rogers, Jennifer T. Wolstenholme, Michael F. Miles

**Affiliations:** ^1^ Department of Pharmacology and Toxicology Virginia Commonwealth University Richmond Virginia USA; ^2^ Program in Neuroscience Virginia Commonwealth University Richmond Virginia USA; ^3^ VCU Alcohol Research Center Virginia Commonwealth University Richmond Virginia USA; ^4^ Department of Human and Molecular Genetics Virginia Commonwealth University Richmond Virginia USA

**Keywords:** alcohol use disorder, ethanol, glycogen synthase kinase 3‐beta, therapeutics, tideglusib, Wnt signalling

## Abstract

Over 10% of the US population over 12 years old meets criteria for alcohol use disorder (AUD), yet few effective, long‐term treatments are currently available. Glycogen synthase kinase 3‐beta (GSK3β) has been implicated in ethanol behaviours and poses as a potential therapeutic target in the treatment of AUD. Here, we investigated the preclinical evidence for tideglusib, a clinically available selective GSK3β inhibitor, in modulating chronic and binge ethanol consumption. Tideglusib decreased ethanol consumption in both a model of daily, progressive ethanol intake (two‐bottle choice, intermittent ethanol access) and binge‐like drinking behaviour (drinking in the dark) without effecting water intake. With drinking in the dark, tideglusib was more potent in males (ED50 = 64.6, CI = 58.9–70.8) than females (ED50 = 79.4, CI = 70.8–93.3). Further, we found tideglusib had no effect on ethanol pharmacokinetics, taste preference or anxiety‐like behaviour, although there was a transient increase in total locomotion following treatment. Additionally, tideglusib treatment did not alter liver function as measured by serum activity of alanine aminotransferase and aspartate aminotransferase but did cause a decrease in serum alkaline phosphatase activity. RNA sequencing analysis of tideglusib actions on ethanol consumption revealed alterations in genes involved in synaptic plasticity and transmission, as well as genes downstream of the canonical Wnt signalling pathway, suggesting tideglusib may modulate ethanol consumption via β‐catenin binding to the transcription factors TCF3 and LEF1. The data presented here further implicate GSK3β in alcohol consumption and support the use of tideglusib as a potential therapeutic in the treatment of AUD.

## Graphical abstract

Tideglusib, a clinically available selective inhibitor of Gsk3b, produced marked decreases in a binge model of ethanol consumption and decreased intake with prolonged intermittent ethanol access. Molecular studies showed tideglusib altered Wnt signalling gene expression in ethanol consuming animals. These results suggest tideglusib is a viable candidate for clinical studies on treatment of alcohol use disorder.

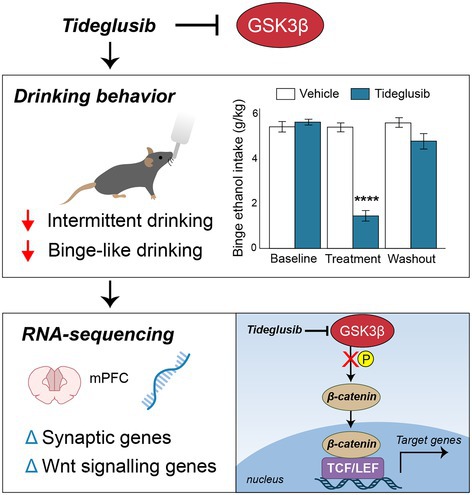

**Abbreviations**
ANOVA: analysis of variance
AUD: alcohol use disorder
DID: drinking in the dark
GSK3β: glycogen synthase kinase 3‐beta
IEA: intermittent ethanol access
PFC: prefrontal cortex

## 1 Introduction

More than 10% of the US population over age 12 are estimated to meet criteria for alcohol use disorder (AUD) [1], with alcohol being the third‐leading cause of preventable death in the United States [2]. Despite these tolls, few treatments for AUD exist, with no new FDA‐approved therapeutic agents within the last 18 years. Additionally, existing treatments are only modestly effective in reducing alcohol consumption and rates of relapse [3].

To elucidate mechanisms underlying the neurobiology of AUD, our laboratory has previously performed genome‐wide expression network profiling of brain regions in mice following acute and chronic ethanol (EtOH) exposure. Those studies reveal the serine–threonine kinase glycogen synthase kinase 3‐beta (GSK3β) to be a hub gene in a network highly regulated by acute EtOH in the medial prefrontal cortex (mPFC) [4, 5, 6] and coanalysis with human genome‐wide association studies identified a GSK3β‐centric network associated with risk for alcohol dependence [6].

GSK3β is ubiquitously expressed throughout the body but has particularly high expression within the brain [7]. Initially discovered for its actions in decreasing glycogen synthesis, GSK3β is now known to be involved in many processes including neuronal differentiation, synapse development, and both short‐ and long‐term synaptic plasticity. GSK3β dysregulation has long been implicated in neurological and psychiatric disorders such as bipolar disorder, schizophrenia, major depressive disorder and Alzheimer's disease [7]. Previous work on substance use disorder revealed cocaine and morphine both activate GSK3β [8, 9], and this activation is required for development of cocaine‐induced conditioned place preference and tolerance to morphine's antinociceptive effects [10, 11].

Prior studies from our laboratory directly implicate GSK3β as a modulator of EtOH‐related behaviours, showing that acute EtOH caused inhibitory phosphorylation of Gsk3b in mPFC while viral‐mediated overexpression of GSK3β within mPFC increases EtOH consumption in male mice. This increase was inhibited by lithium, a drug that pharmacologically inhibits both the alpha and beta paralogs of GSK3. Additionally, in the same report our laboratory showed that the more selective GSK3β inhibitor TDZD‐8 decreases operant self‐administration of EtOH in rats [6].

Tideglusib, also known as NP031112 or NP‐12, is a highly selective, non‐ATP competitive, GSK3β inhibitor that has greater bioavailability than its parent compound, TDZD‐8 [12]. Tideglusib has also been studied in clinical trials for several neurodegenerative and developmental disorders [13, 14, 15, 16, 17]. Here, we report actions of tideglusib on EtOH consumption in both a prolonged two‐bottle choice, intermittent ethanol access (IEA) and a drinking in the dark (DID) model and assess potential toxicity through responses in other behaviours and liver functions to further assess tideglusib's potential as a therapeutic agent for AUD. We additionally investigate mechanisms of tideglusib action on EtOH genomic responses to implicate GSK3β targets involved in synaptic plasticity and neurotransmission. Our findings further support GSK3β inhibitors such as tideglusib as candidate therapeutics in AUD.

## 2 Materials and Methods

### 2.1 Animals

Rodent animal studies and procedures were approved by the Institutional Animal Care and Use Committee of Virginia Commonwealth University and followed the NIH Guide for the Care and Use of Laboratory Animals. Male and female C57BL/6J mice, purchased from Jackson Laboratories (Bar Harbor, Maine), were used for all studies, with the exception of IEA Experiment 2 and blood ethanol content (BEC) studies which utilised only males. Mice were housed in a temperature and humidity‐controlled room in accordance with the Association for Assessment and Accreditation of Laboratory Animal Care–approved animal care facility and had ad libitum access to food and water. Animals were housed on Tekland Laboratory Grade Sani‐Chips bedding.

### 2.2 Drugs

EtOH for drinking studies was prepared as a 20% (v/v) solution in tap water and provided to animals for voluntary oral consumption. For other studies utilising EtOH, it was made up as a 20% (v/v) solution in 0.9% saline and delivered via intraperitoneal (i.p.) injection at a dose of either 1.8 or 2.0 g/kg dependent on experiment. The GSK3β inhibitor tideglusib (Selleck Chemicals, Houston, Texas) was prepared for gavage by suspension at 20 mg/mL in 26% peg‐400 (Sigma) and 15% Cremophor EL (Sigma) in water for IEA Experiment 1 and BEC studies. Tideglusib was prepared in corn oil to increase ease of suspension for all other studies at 20 mg/mL with the exception of DID where concentrations were varied to maintain a consistent injection volume across all groups. A dose of 200 mg/kg was used in initial IEA Experiment 1 and for studies of tideglusib effects on EtOH pharmacokinetics, taste preference or locomotor activity (Exp. 6–8). Other experiments used 100 mg/kg per injection except DID studies dose–response analysis ranging from 0 to 100 mg/kg. Tideglusib was suspended in vehicle by vortexing followed by bath sonication at 40°C for 60 min. Tideglusib injection or gavage volumes were ~10 mL/kg or ~0.25 mL/animal.

### 2.3 IEA, Two‐Bottle Choice

**FIGURE 1:**
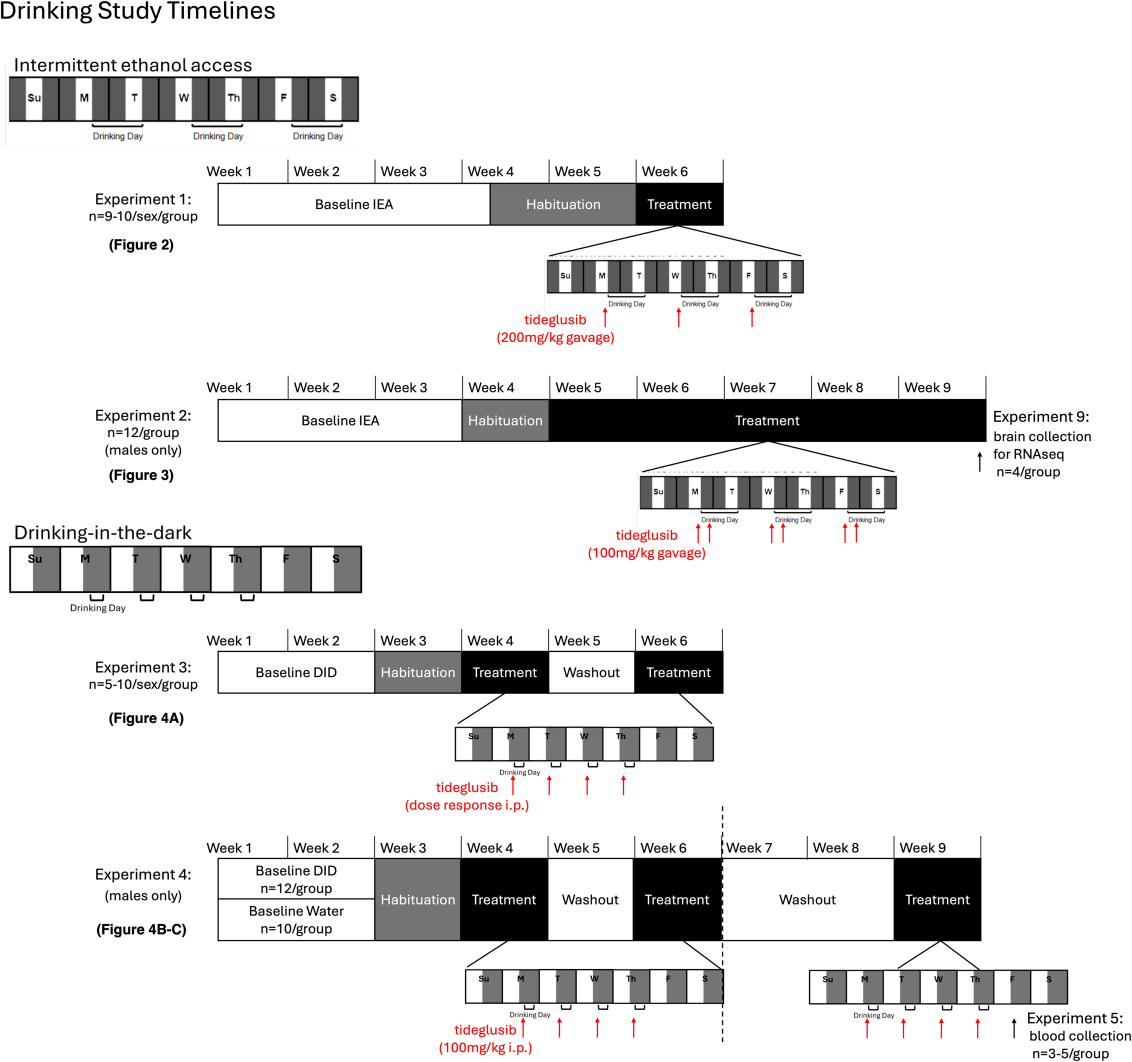
Schematic representation of the timelines for all drinking studies. Red arrows indicate when tideglusib was administered. Black arrows indicate collection point (brain, blood) for given experiment. Experimental protocols correspondent to specific figures are indicated.

A schematic of the timeline for all EtOH drinking studies is shown in Figure 1.

Experiment 1: Male and female mice (*n* = 9–10/group) underwent two‐bottle choice, IEA for 6 weeks with minor modifications from our prior published method [18]. Mice had access to two water drinking bottles. On Mondays, Wednesdays and Fridays, one of the water bottles was replaced with 20% EtOH v/v at the beginning of the dark cycle and left on for 24 h, with sides alternated each drinking session to avoid conditioning a side preference. Two hours (binge reading) and 24 h after access to EtOH, volume measurements were taken, and EtOH consumption (gramme/kilogramme), preference over water and total fluid consumption (millilitre) were calculated. Baseline IEA consumption values (gramme/kilogramme) were measured for 3 weeks. Mice were then acclimated to gavage treatments with 3‐day saccharin administration followed by 2‐day vehicle habituation before beginning gavage of 200 mg/kg tideglusib in peg‐400/Cremophor (0.01 mL/g body weight) or equivalent volume of vehicle on Week 6, after which effects on consumption and preference were measured for the week. Gavage treatments occurred 30–60 min prior to EtOH access. Due to a small number of isolated animals showing mild weight loss and to aid more efficient drug suspension, vehicle for tideglusib experiments was changed to corn oil following completion of Experiments 1 and 7.

Experiment 2: To allow for greater power within groups and due to the number of treatment groups, only male mice were included (*n* = 12/group). A total of four treatment groups were included with two drinking groups (water only vs. EtOH IEA) and two drug treatment groups (tideglusib vs. vehicle), to give a total of 48 animals. A subgroup of these animals (*n* = 4/group) was used for RNAseq studies in Experiment 9 as below. Mice underwent the same two‐bottle choice, IEA paradigm as in Experiment 1 but for a total of 9 weeks. This timeline was extended from the previous IEA experiment in order to study actions of tideglusib over a longer period of administration and assess for any signs of habituation to its effects on EtOH consumption. Additionally, the habituation phase was shortened as there was no significant difference in consumption values between 2 and 3‐day saccharin habituation. Water‐drinking animals were treated identically to the IEA groups. Baseline IEA consumption values (gramme/kilogramme) were measured for 3 weeks. Mice were then acclimated to gavage treatments with 2‐day saccharin administration followed by 2‐day vehicle habituation before beginning gavage of 100 mg/kg tideglusib or equivalent volume vehicle on Week 5, after which effects on consumption, preference and total fluid intake were measured for four additional weeks. Two separate gavage treatments were administered each drinking day at 2 h prior to EtOH access and 4 h later, occurring immediately after the 2‐hour binge read.

### 2.4 DID

Experiment 3: For binge EtOH studies using the DID model, male and female C57BL/6J mice (*n* = 20/sex) were given access to 20% v/v EtOH for 4 h as a single bottle access with no water present, 4 days a week, beginning 3 h after lights off from Monday to Thursday. At the conclusion of the 4‐hour test, EtOH was removed and replaced with water bottles until the next experimental day. Baseline DID consumption values (gramme/kilogramme/4 h) were measured for 2 weeks. An i.p. route of tideglusib administration was selected for DID studies to allow for a more rapid onset of drug effects compared to oral gavage given the limited 4‐h drinking sessions compared to the 24‐h IEA studies. Mice were then acclimated to i.p. injections with 2 days saline and 4 days corn oil before beginning injections of tideglusib. The first cycle of DID studies included doses of 10–100 mg/kg tideglusib injected i.p. 1 h prior to EtOH access. Following a week of drug washout, groups were rerandomised and a second cycle of tideglusib was administered at 50–100 mg/kg. Controls had corn oil vehicle injections. Final group sizes were *n* = 10/sex for control and 100 mg/kg doses, and *n* = 5/sex for all other doses.

Experiment 4: As the 100 mg/kg dose was most effective in both sexes and decreased consumption to similar levels in the two, this dose was used in additional DID studies. To assess time until washout of drug effects, a separate set of animals (*n* = 24, males) underwent DID as described above. Mice received 100 mg/kg tideglusib or corn oil vehicle via i.p. injection 1 h prior to EtOH access (*n* = 12/group) for 1 week. Mice then had an additional week of continued access to DID EtOH without any further tideglusib injections. Additionally, another group of mice (*n* = 10/group, males) underwent this same DID testing except with a single bottle access to water during the testing period to assess possible tideglusib action on fluid consumption in general.

### 2.5 Liver Function Assessment

Experiment 5: Blood serum was collected from male mice (*n* = 3–5/group) following 9 weeks of water or EtOH DID from Experiment 4. During the last week of DID, animals received i.p. injections of 100 mg/kg tideglusib or corn oil vehicle, and 24 h after their last injection, blood was collected retro‐orbitally. Serum was isolated and assessed for a panel of liver enzymes as a measure of liver functions, including alanine aminotransferase (units/litre), aspartate aminotransferase (units/litre) and alkaline phosphatase (units/litre), using Vetscan preventative care profile plus kits (Zoetis US, Parsippany, New Jersey).

### 2.6 BEC

Experiment 6: Male mice (*n* = 25) were gavaged with tideglusib (200 mg/kg) or 26% peg‐400 (Sigma), 15% Cremophor EL (Sigma) in water vehicle and 30 min later i.p. injected with 2.0 g/kg EtOH. These higher doses of tideglusib were used to fully assess effects on pharmacokinetics, taste preference or off‐target effects on locomotion (Experiments 6–8). Blood was collected via submandibular cheek punch at 10, 30, 60 and 90‐min time points post‐EtOH (*n* = 3–4/treatment/time). Blood was collected in BD microtainer tubes containing EDTA (Fisher Scientific, Waltham, Massachusetts) and stored at −20°C until analysis. BECs were assessed by the VCU Analytics Core using headspace gas chromatography [19]. EtOH content was calculated based on normalisation to a consistent internal standard of 1‐proponal in each sample and reported as milligram/litre.

### 2.7 Taste Preference

Experiment 7: Male and female mice (*n* = 7–8/group) were gavaged with 200 mg/kg tideglusib or vehicle for 6 days. They were given access to two bottles containing water and either saccharin or quinine 1 h after tideglusib administration. Saccharin and quinine concentrations were 0.6 mM and 25 μM respectively for Days 1–3 and were increased to 1.2 mM and 40 μm for Days 4–6. Quinine or saccharin preference compared to water was measured daily.

### 2.8 Light/Dark Box

Experiment 8: Two to 3 weeks prior to undergoing taste preference testing, the same male and female mice from Experiment 7 (*n* = 4–8/group) were gavaged with 200 mg/kg tideglusib or corn oil and 30 min later i.p. injected with 1.8 g/kg 20% v/v EtOH in saline or equivalent volume saline vehicle and put into a cage by themselves. Five minutes postinjection, mice were placed into the light side of light/dark boxes (Med Associates, Fairfax, Vermont) facing the dark compartment and assayed for 10 min. Percent time in light, percent distance travelled in light and total locomotion were measured. The duration of the test was separated into two consecutive 5‐min time bins.

### 2.9 RNA Sequencing Analysis

Experiment 9: A subset of mice from Experiment 2 (*n* = 4/group × 2 drinking groups × 2 drug treatments) were euthanised 24 h after their last EtOH access for RNAseq analysis of gene expression in PFC and nucleus accumbens. Total RNA was isolated from dissected tissue and sent for sequencing at the VCU Genomics Core. For purposes of evaluation of tideglusib actions on EtOH consumption in this manuscript, only analysis between EtOH‐drinking, tideglusib‐treated mice and EtOH‐drinking, vehicle‐treated mice in PFC is reported due to the overall scope and complexity of the entire RNAseq dataset. Full details are included in Supporting Information and detailed reporting of RNAseq data will be presented elsewhere (Gottlieb et al., manuscript in preparation) and are published as a preprint manuscript [20]. All results of paired differential expression data are also available on the Open Science Framework (https://osf.io/g7pmn/?view_only=3345a35f2cb94c9da0cf1641512dbf04). Paired end counts were analysed for differential expression analysis across treatment groups using DESeq2 [21]. Genes with median counts < 1 across all samples were filtered out of the data, and significantly differently expressed genes (DEGs) were determined using an uncorrected *p* < 0.05 and a log‐fold change (LFC) of |LFC| > 0.1. Gene ontology analysis of DEGs was performed using ToppGene [22].

### 2.10 Statistical Analysis

All statistical tests were performed in R with the exception of linear regression and 50% effective dose (ED50) calculations which were performed in GraphPad Prism. A *p* value of 0.05 or lower was considered statistically significant. The exact statistical test performed is documented in the Results section for each experiment.

## 3 Results

### 3.1 A Single Dose of 200 mg/kg Tideglusib Decreases EtOH Consumption During Two‐Bottle Choice, IEA Binge Drinking

**FIGURE 2:**
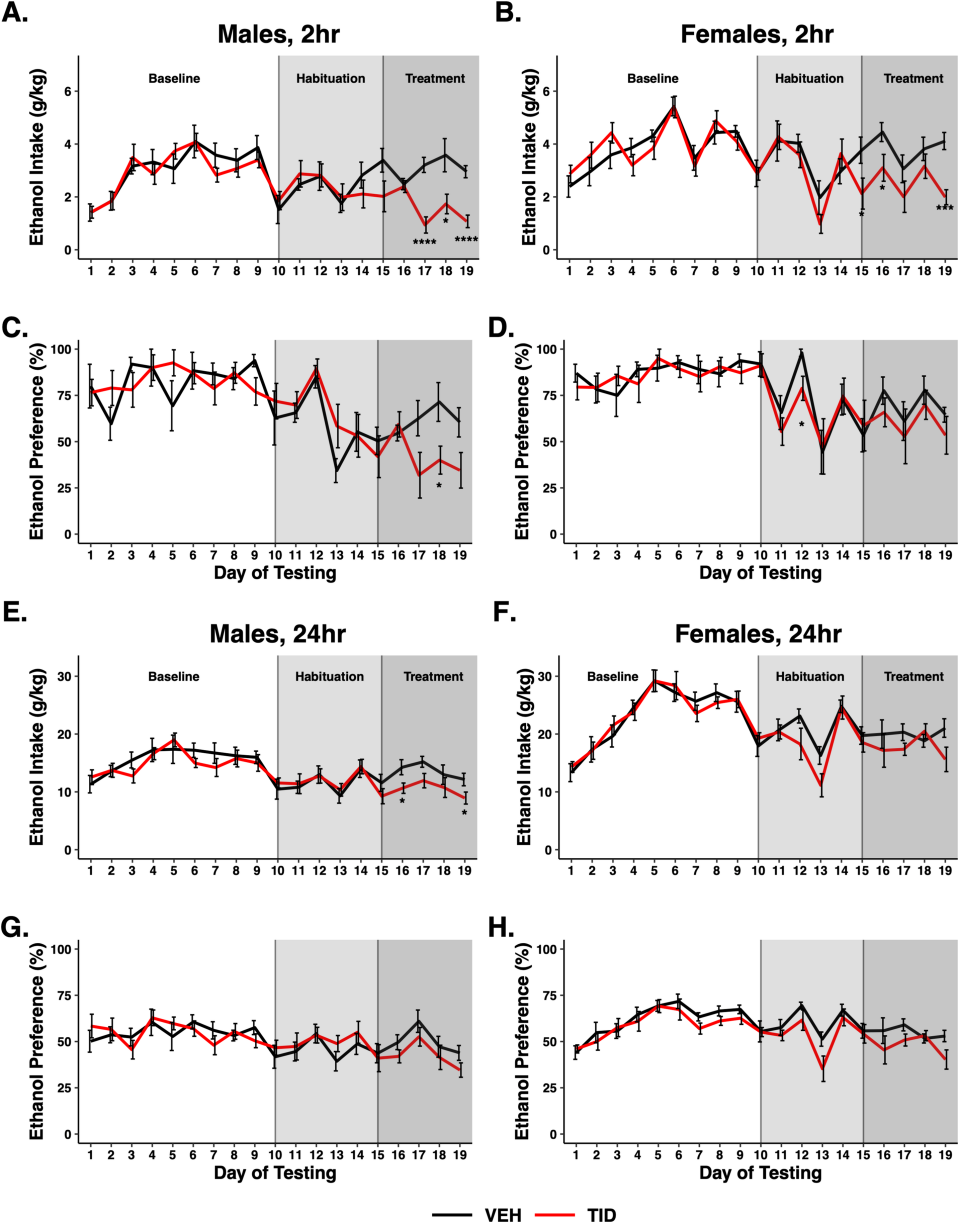
A single 200 mg/kg dose of tideglusib decreased binge ethanol consumption. Results are shown for binge (2 h, A–D) and daily (24 h, E–H) consumption or preference in male and female animals. Compared to vehicle‐treated mice, tideglusib‐treated mice (*n* = 9–10/group) showed (A) a main effect of treatment day on decreased binge ethanol consumption (*F*_18,288_ = 6.555, *p* < 0.0001) and a treatment group*treatment day interaction (*F*_18,288_ = 2.066, *p* = 0.007) in males and (B) a main effect of treatment day on decreased binge ethanol consumption (*F*_18,306_ = 9.099, *p* < 0.0001) and a treatment group*treatment day interaction (*F*_18,306_ = 1.839, *p* = 0.02) in females. Bonferroni post hoc analysis showed significant differences between treatment groups on individual days (*p < 0.05, ***p < 0.001, ^****^ *p* < 0.0001) for binge consumption in both males and females. There was a main effect of treatment day on binge ethanol preference (C, D) in both males (*F*_18,216_ = 6.789, *p* < 0.0001) and females (*F*_18,234_ = 4.683, *p* < 0.0001) but no effect of tideglusib or tideglusib interaction. There was an effect of treatment day on daily consumption (E, F) in both males (*F*_18,252_ = 7.363, *p* < 0.0001) and females (*F*_18,234_ = 14.853, *p* < 0.0001) and preference (G, H) in both males (*F*_18,252_ = 3.337, *p* < 0.0001) and females (*F*_18,216_ = 8.183, *p* < 0.0001) but no effect of tideglusib treatment or a tideglusib interaction on any daily measure.

Experiment 1: The goal of this initial study was to determine if a single high oral dose (200 mg/kg) of tideglusib would decrease EtOH consumption at two time points, 2 h (binge, Figure 2A–D) and 24 h (daily, Figure 2E–H) in male and female animals. Prior to tideglusib administration, mice escalated their drinking during 3 weeks of baseline IEA consumption as expected (Figure 2A–F), particularly with daily consumption in females. Initial analysis of tideglusib effects consisted of a three‐way repeated measures (RM) ANOVA with sex, treatment day and treatment group as variables. For binge consumption, there was, as expected, a main effect of sex on EtOH consumption at the binge timepoint (*F*_1,33_ = 18.736, *p* = 0.0001) and a main effect of day (*F*_18,594_ = 13.96, *p* = 3.44E−35) but no significant main effect of treatment (*F*_1,33_ = 3.073, *p* = 0.089) or treatment*sex interaction (*p* = 0.997). Daily consumption showed similar results with a main effect of sex (*F*_1,27_ = 52.144, *p* = 9.12E−8), treatment day (*F*_8.7,234.9_ = 20.69, *p* = 5.87E−25) and sex*treatment day (*F*_8.7,234.9_ = 3.34, *p* = 8.45E−4) but no significant main effect of tideglusib (*F*_1,27_ = 3.963, *p* = 0.057), treatment*sex interaction (*F*_1,27_ = 0.279, *p* = 0.602) or any other interaction with treatment. Since it is very well established that female C57BL/6J mice consume more EtOH than males [23, 24, 25, 26, 27], we proceeded with this as an a priori assumption and analysed sexes separately in all studies moving forward.

When sexes were separated, tideglusib‐treated mice consumed significantly less EtOH than their vehicle‐treated counterparts in both males and females at the binge EtOH timepoint. Two‐way RM ANOVA revealed a main effect of treatment day (*F*_18,288_ = 6.555, *p* < 0.0001) and a treatment group*treatment day interaction (*F*_18,288_ = 2.066, *p* = 0.007) in male mice on binge consumption (Figure 2A) and a main effect of treatment day (*F*_18,216_ = 6.789, *p* < 0.0001) on binge EtOH preference (Figure 2C). Bonferroni post hoc analysis yielded significant differences in binge consumption on Days 17–19 and binge preference on Day 18 in tideglusib versus vehicle mice (*p* < 0.05).

Female mice showed a main effect of treatment day (*F*_18,306_ = 9.099, *p* < 0.0001), a treatment group*treatment day interaction (*F*_18,306_ = 1.839, *p* = 0.02) on binge consumption (Figure 2B) and a main effect of treatment day (*F*_18,234_ = 4.683, *p* < 0.0001) on binge preference (Figure 2D). Bonferroni post hoc analysis highlighted significant differences in binge consumption on Days 15–16 and 19 and binge preference on Day 12 in tideglusib versus vehicle mice (*p* < 0.05).

The single‐dose tideglusib paradigm failed to elicit a main effect or interaction of tideglusib on EtOH 24‐h drinking behaviours; however, there was a main effect of day in both male consumption (*F*_18,252_ = 7.363, *p* < 0.0001) and preference (*F*_18,252_ = 3.337, *p* < 0.0001) and female consumption (*F*_18,234_ = 14.853, *p* < 0.0001) and preference (*F*_18,216_ = 8.183, *p* < 0.0001). Bonferroni post hoc analysis did reveal significant treatment effects in male daily consumption on Days 16 and 19 (*p* < 0.05) but no differences in females (Figure 2E–H).

Additionally, body weights were averaged over the pre‐ and posttideglusib treatment phases and tideglusib‐treated mice were compared to vehicle controls as a proxy measurement for caloric intake during the study. There was a main effect of treatment phase (*F*_1,35_ = 54.003, *p* < 0.0001) as the animals naturally gained weight over time and an effect of sex where males were larger than females (*F*_1,35_ = 273.621, *p* < 0.0001) but no effect of tideglusib nor any tideglusib interaction, suggesting tideglusib was not altering EtOH intake through caloric regulation in either male or female animals (Figure S1A).

### 3.2 Two Daily 100 mg/kg Doses of Tideglusib Decrease EtOH Consumption and Preference During Two‐Bottle Choice, IEA Binge and Daily Drinking in Male Mice

**FIGURE 3:**
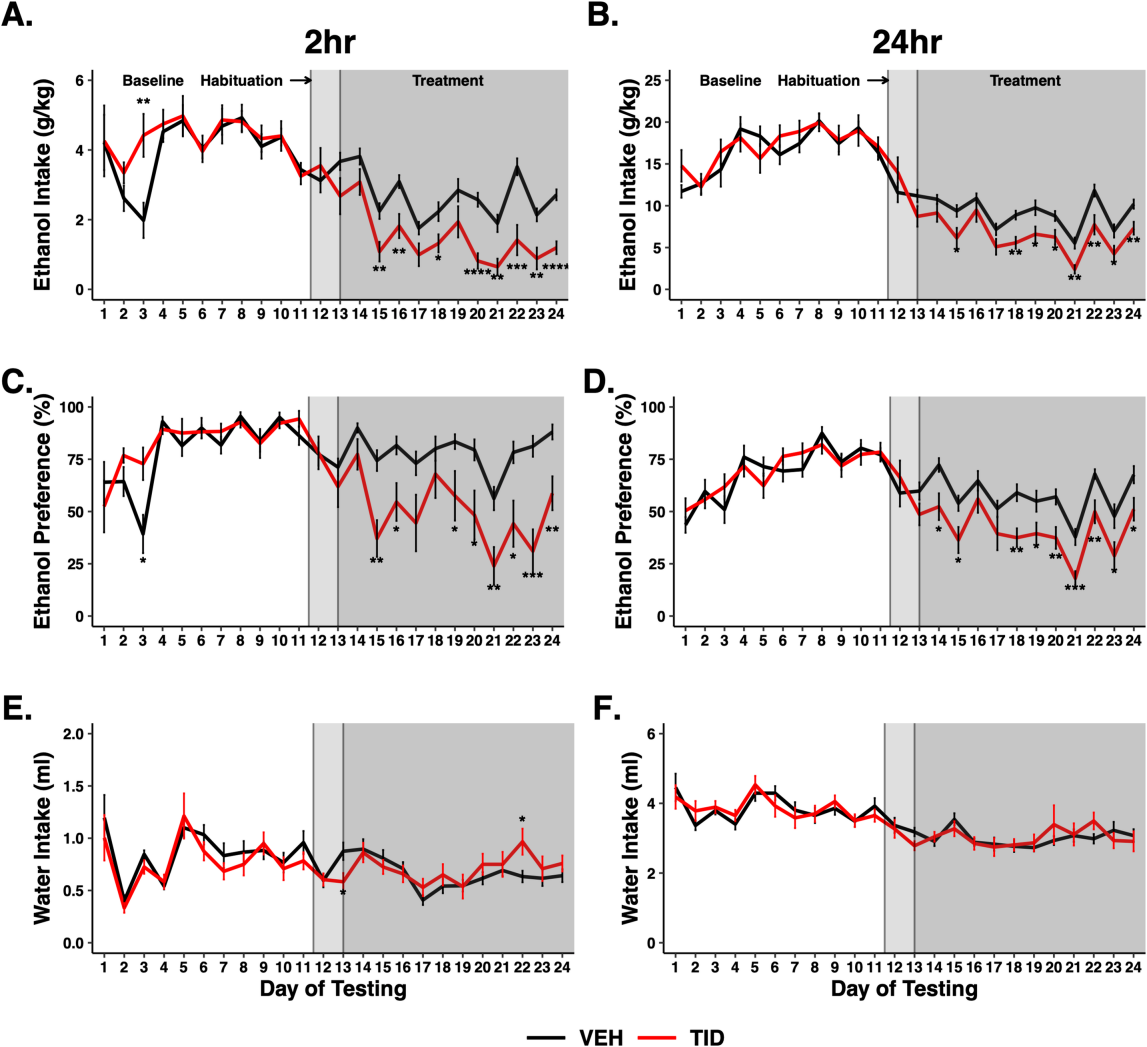
Two separate 100 mg/kg doses of tideglusib decreased binge and daily ethanol consumption and preference in males. Compared to vehicle‐treated mice, tideglusib‐treated mice (*n* = 12/group, males) showed (A) a main effect of tideglusib treatment (*F*_1,22_ = 5.421, *p* = 0.029), an effect of treatment day (F_23,506_ = 21.499, p < 0.001), and a treatment group*treatment day interaction (*F*_23,506_ = 3.448, *p* < 0.001) on binge ethanol consumption and (B) an effect of treatment day (*F*_23,483_ = 48.045, *p* < 0.001) and a treatment group*treatment day interaction (*F*_23,483_ = 2.128, *p* = 0.002) on daily ethanol consumption. (C) There was a main effect of treatment group (*F*_1,22_ = 11.420, *p* = 0.003), treatment day (*F*_23,506_ = 9.544, *p* < 0.001) and treatment group*treatment day interaction (*F*_23,506_ = 4.205, *p* < 0.001) in binge ethanol preference and (D) an effect of treatment group (*F*_1,21_ = 5.254, *p* = 0.032), treatment day (*F*_23,483_ = 19.249, *p* < 0.001) and treatment group*treatment day interaction (*F*_23,483_ = 2.527, *p* < 0.001) on daily ethanol preference. Bonferroni post hoc analysis showed significant differences between treatment groups on individual days (*p < 0.05, **p < 0.01, ***p < 0.001, ^****^ *p* < 0.0001). (E) There was a main effect of treatment day on water intake in both the binge (*F*_23,506_ = 7.395, *p* > 0.0001) and (F) daily timepoints (*F*_23,483_ = 11.680, *p* < 0.0001) but no effect of tideglusib treatment nor interaction.

Experiment 2: This experiment tested whether twice daily administration of tideglusib would impact 24‐h EtOH consumption and do so over a prolonged period of time (9 weeks) without evidence of tachyphylaxis. Additionally, animals from this experiment were utilised for RNAseq analysis as below. Due to the length and complexity of the experimental design, only male animals were studied so as to achieve adequate power. Prior to tideglusib administration, mice escalated their drinking during the 4 weeks of baseline, with no difference between groups. For the 2‐h binge reading, two‐way RM ANOVA showed a main effect of treatment group (*F*_1,22_ = 5.421, *p* = 0.029), treatment day (*F*_23,506_ = 21.499, *p* < 0.001) and treatment group*treatment day interaction (*F*_23,506_ = 3.448, *p* < 0.001) on consumption (Figure 3A) and a main effect of treatment group (*F*_1,22_ = 11.420, *p* = 0.003), treatment day (*F*_23,506_ = 9.544, *p* < 0.001) and treatment group*treatment day interaction (*F*_23,506_ = 4.205, *p* < 0.001) on preference (Figure 3C). Bonferroni post hoc analysis revealed significant differences between treatment groups during Day 3 of baseline drinking and Days 15–16, 18 and 20–24 during tideglusib treatment (*p* < 0.05) for consumption and Days 3, 15–16 and 19–24 for preference (*p* < 0.05).

Tideglusib also significantly reduced intake and preference at 24 h under this dosing paradigm, with two‐way RM ANOVA revealing an effect of treatment day (*F*_23,483_ = 48.045, *p* < 0.001) and a significant treatment group*treatment day interaction (*F*_23,483_ = 2.128, *p* = 0.002) on consumption (Figure 3B) and an effect of treatment group (*F*_1,21_ = 5.254, *p* = 0.032), treatment day (*F*_23,483_ = 19.249, *p* < 0.001) and treatment group*treatment day interaction (*F*_23,483_ = 2.527, *p* < 0.001) on preference (Figure 3B). Bonferroni post hoc analysis yielded significant differences between treatment groups during Day 3 of baseline drinking and Days 15 and 18–24 (*p* < 0.05) for consumption and Days 14–15 and 18–24 during tideglusib treatment for preference (*p* < 0.05).

To assess effects of tideglusib on water consumption (and as a control in RNAseq studies), an additional group of EtOH‐naïve mice (*n* = 12) receiving only two bottles of water was run concurrently throughout the course of the experiment. There was a main effect of treatment day on water intake in both the binge (*F*_23,506_ = 7.395, *p* < 0.0001) and daily timepoints (*F*_23,483_ = 11.680, *p* < 0.0001) but no effect of tideglusib treatment (Figure 3E,F), suggesting the actions of tideglusib are specific to decreasing EtOH intake and not overall fluid intake in our model. Additionally, body weights were averaged over the pre‐ and posttideglusib treatment phases and tideglusib‐treated mice were compared to vehicle controls. There was a main effect of treatment phase (*F*_1,44_ = 247.912, *p* < 0.0001) but no effect of EtOH, tideglusib, nor any interactions, again suggesting tideglusib is not altering EtOH intake through caloric regulation (Figure S1B).

### 3.3 Tideglusib Treatment Decreases EtOH Consumption During DID

**FIGURE 4:**
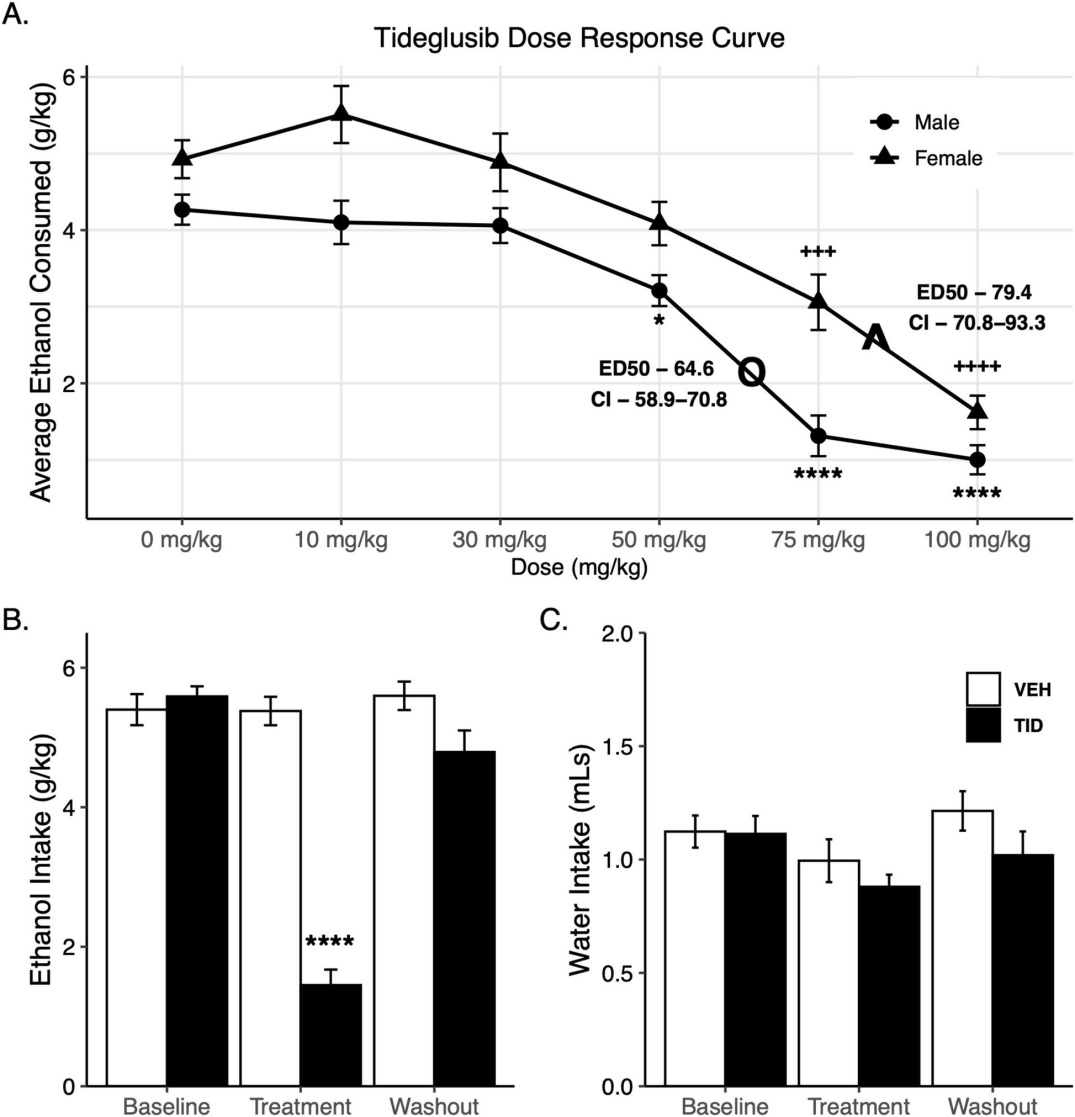
Tideglusib decreased ethanol consumption during drinking in the dark without affecting water intake. (A) Tideglusib treatment decreased ethanol consumption in a dose‐dependent manner (*F*_5,67_ = 61.215, *p* < 0.0001). Females consumed more gramme/kilogramme EtOH than males over the course of the study (*F*_1,67_ = 35.565, *p* < 0.0001) (*n* = 10–20/sex/dose). There was no sex*treatment interaction. * denotes significance between dose and vehicle control in males and + denotes significance in females (*p < 0.05, ^+++^ *p* < 0.001, ^****/++++^ *p* < 0.0001). Males had a lower ED50 (oED50 = 64.6, CI = 58.9–70.8) than females (^ED50 = 79.4, CI = 70.8–93.3) and 95% CI do not overlap, showing tideglusib is significantly more potent in males. (B) 100 mg/kg of tideglusib significantly decreased ethanol consumption dependent on treatment phase (baseline, treatment, washout) (*F*_2,59_ = 61.60, *p* > 0.0001) and treatment group (*F*_1,59_ = 59.73, *p* > 0.0001) and shows a phase*treatment interaction (*F*_2,59_ = 43.45, *p* > 0.0001). Tideglusib decreased consumption during the treatment phase compared to baseline consumption and drug washout and is significantly different from vehicle treatment during the treatment period (^****^ *p* < 0.0001). (C) Tideglusib treatment has no effect on water intake.

Experiment 3: To determine if tideglusib was also effective in modulating EtOH intake in a model of binge EtOH consumption, we conducted a dose–response study using male and female animals in the DID drinking paradigm. Tideglusib was administered by i.p. injections for these studies due to the short time course of daily EtOH consumption (4 h). There was an effect of sex during DID (*F*_1,67_ = 35.565, *p* < 0.0001) where females consumed more gramme/kilogramme EtOH than males, as well as an effect of tideglusib dose (*F*_5,67_ = 61.215, *p* < 0.0001) according to two‐way ANOVA. Bonferroni post hoc analysis revealed that doses of 50 mg/kg (*p* = 0.017), 75 mg/kg (*p* < 0.0001) or 100 mg/kg (*p* < 0.0001) all decreased consumption in male mice compared to a vehicle control. There was no further decrease in consumption with 100 mg/kg compared to 75 mg/kg. In females, a dose of 75 mg/kg (*p* = 0.0002) or 100 mg/kg (*p* < 0.0001) significantly decreased consumption compared to vehicle; however, the higher dose of 100 mg/kg was significantly different from the 75 mg/kg dose (*p* = 0.009) (Figure 4A). Tideglusib doses were converted to logarithmic doses to show linear regression and calculate ED50 values in both males and females. *T*‐test showed no difference between males and females at the 0 mg/kg dose (*p* = 0.11), and ED50s were calculated based off this 0 mg/kg baseline response. The 10 mg/kg dose was removed from the regression as it was statistically no different from the 30 mg/kg dose. The ED50 values and 95% confidence intervals (CIs) were calculated by GraphPad Prism in log dose units and then converted back to linear units for ease of communication. The ED50 was lower in males (ED50 = 64.6, CI = 58.9–70.8) than females (ED50 = 79.4, CI = 70.8–93.3), and 95% confidence limits did not overlap, showing tideglusib to be slightly more potent in males than females (Figure S2).

Experiment 4: In order to evaluate whether tideglusib effects on EtOH consumption were reversable, we conducted a drug washout analysis. A separate cohort of male mice was run in the DID model using the most effective dose from both sexes (100 mg/kg), and two‐way ANOVA revealed a significant effect of experimental phase (baseline, treatment, washout) (*F*_2,59_ = 61.60, *p* > 0.0001), tideglusib treatment (*F*_1,59_ = 59.73, *p* > 0.0001) and a phase*treatment interaction (*F*_2,59_ = 43.45, *p* > 0.0001). Prior to tideglusib treatment, mice assigned to either treatment group consumed equal amounts of EtOH in DID. Tideglusib significantly decreased EtOH consumption compared to baseline values, and tideglusib‐treated mice drank significantly less than vehicle‐treated mice during the treatment period (*p* < 0.0001). Following washout, tideglusib‐treated mice returned to baseline consumption values, and there was no significant difference between drug versus vehicle‐treated animals during washout (Figure 4B). Tideglusib had no effect on water intake (Figure 4C).

### 3.4 Tideglusib Treatment Has No Effects on Blood EtOH Pharmacokinetics, Taste Preference or EtOH‐Induced Anxiolysis and Modest Effects on Serum Alkaline Phosphatase Levels and Locomotion

Experiment 5: To test for potential liver toxicity of tideglusib in the absence or presence of EtOH, we assessed liver functions by serum enzyme levels following 9 weeks of DID water or EtOH drinking (36 drinking days) with the last week having treatment +/− tideglusib × 4 days (100 mg/kg). Two‐way ANOVA showed there was no effect of EtOH, tideglusib or EtOH + tideglusib treatment on levels of alanine aminotransferase (Figure S3B) or aspartate aminotransferase (Figure S3C). However, tideglusib treatment did decrease levels of alkaline phosphatase (*F*_1,12_ = 20.018, *p* = 0.0008) (Figure S3D). Tukey's HSD post hoc revealed a significant difference in alkaline phosphatase activity between water‐drinking animals treated with tideglusib versus vehicle (*p* = 0.0478) and between EtOH‐drinking tideglusib versus vehicle (*p* = 0.0249).

Experiment 6: Pharmacokinetic analysis showed there was an expected significant effect of time following EtOH injection on BECs according to two‐way ANOVA (*F*_3,18_ = 12.345, *p* < 0.0001). There was no effect of tideglusib treatment (200 mg/kg) compared to vehicle controls nor a treatment*time interaction, suggesting tideglusib does not alter EtOH pharmacokinetics (Figure S3A).

**FIGURE 5:**
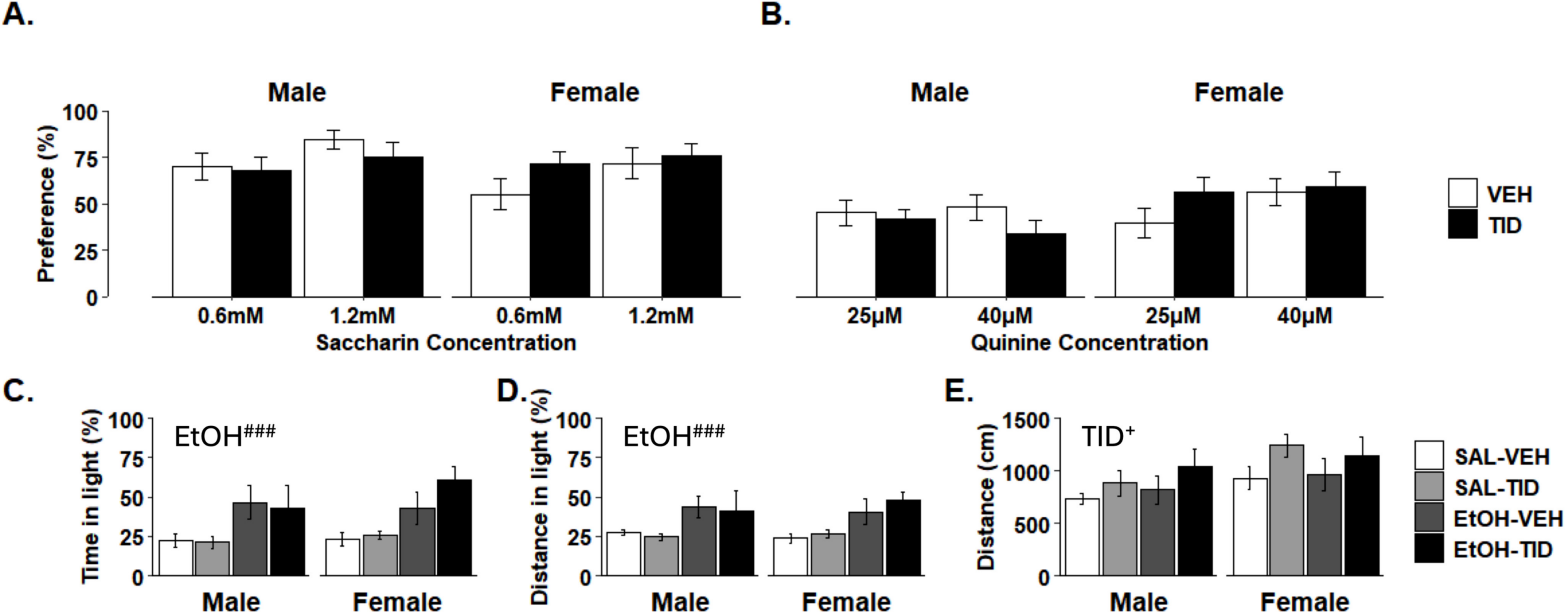
Tideglusib has no effect on taste preference or ethanol‐induced anxiety but transiently increases total locomotion. (A) Tideglusib treatment does not change taste preference in either sex (*n* = 7–8/group) for saccharin at 0.6 or 1.2 mM (B) nor quinine at 25 or 40 μM concentrations. (C, D) i.p. ethanol significantly decreases anxiety‐like behaviour in the light–dark box as measured by percent time in light (*F*_1,50_ = 19.172, ^###^ *p* < 0.001) and (B) percent distance in light (*F*_1,50_ = 17.264, *p* = 0.0001) with no effect of sex or tideglusib (*n* = 4–8/group). (E) Tideglusib transiently increases total locomotion in the light–dark box (*F*_1,50_ = 4.167, +*p* = 0.046); however, no comparisons reached significance in post hoc testing.

Experiment 7: Tideglusib (200 mg/kg) also did not alter taste preference for quinine nor saccharin at either the low or high concentrations tested according to two‐way ANOVAs run for each sex and taste separately (Figure 5A,B).

Experiment 8: In the light/dark box, there was a main effect of EtOH on anxiety‐like behaviours as measured by a three‐way ANOVA in percent time in light (*F*_1,50_ = 19.172, *p* < 0.001) and percent distance in light (*F*_1,50_ = 17.264, *p* = 0.0001), where EtOH treatment produced significant EtOH‐induced anxiolysis. However, tideglusib treatment (200 mg/kg) did not alter anxiety‐like behaviour as measured by either percent time (Figure 5C) or percent distance in light (Figure 5D), nor was there a treatment*EtOH interaction. Tideglusib did transiently increase locomotion during the first 5 min of the assay (*F*_1,50_ = 4.167, *p* = 0.047) (Figure 5E), but there was no effect during the second 5 min of the assay, nor when the entire 10‐min test was compared (Figure S4). There was no effect of sex on any measure.

### 3.5 Tideglusib Treatment Regulates Genes Involved in Synaptic Signalling, Plasticity and the Wnt Signalling Pathway

**FIGURE 6:**
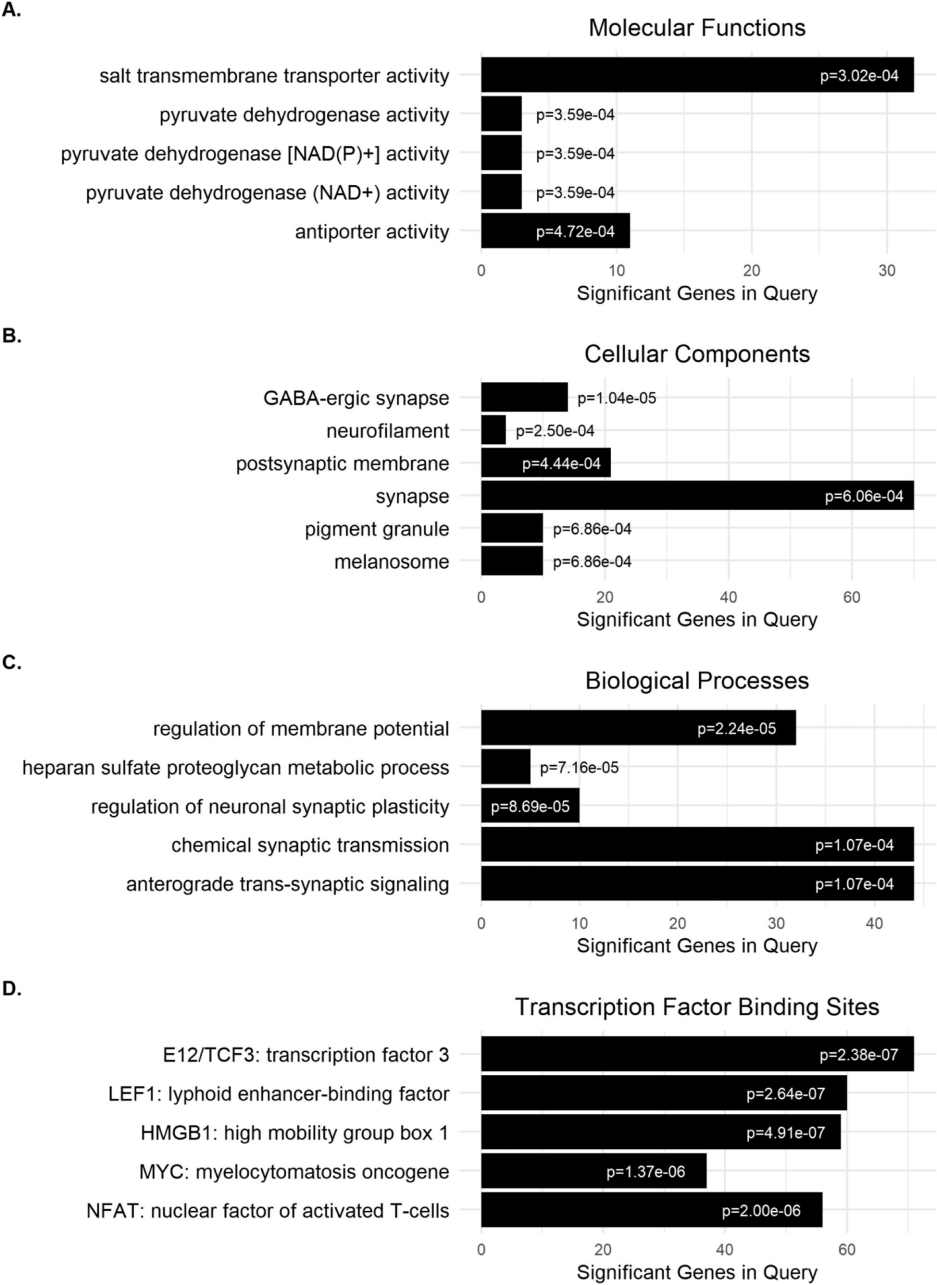
Tideglusib‐treated mice show differential gene expression in the mPFC across multiple gene ontology categories. The top five gene ontologies for the following categories in ethanol‐drinking mice treated with tideglusib versus vehicle (*n* = 4/group) and their significant *p* values are shown. (A) Molecular functions, (B) cellular components, (C) biological processes and (D) transcription factor binding sites. All five TFBS ontology results are involved in Wnt signalling, with TCF3 and LEF1 binding β‐catenin as part of the canonical Wnt signalling pathway.

Experiment 9: As an initial approach to assess mechanisms of tideglusib action in decreasing EtOH consumption, we performed a detailed RNAseq analysis on a subset of animals from Experiment 2. Full details of that analysis will be reported elsewhere (Gottlieb et al., manuscript in preparation) and are available as a preprint publication [20]. Here, we present results from PFC comparing EtOH‐drinking animals treated with vehicle versus tideglusib to contribute to the mechanism of drug action in the presence of EtOH. RNAseq differential expression analysis revealed 711 DEGs in the PFC of male mice between EtOH drinking animals treated with tideglusib versus vehicle using cutoffs of *p* < 0.05 and |LFC| > 0.1 (Table S1). Of these 711 DEGs, only 13 genes overlapped with DEGs from water drinking animals treated with tideglusib alone versus vehicle (see https://osf.io/g7pmn/?view_only=3345a35f2cb94c9da0cf1641512dbf04). Analysis of DEGs by gene ontology in ToppGene revealed highly significant functional group overrepresentation across multiple categories, including molecular functions, cellular components, biological processes and transcription factor binding sites (Table S2). The top five ontology results in each of these categories showed involvement in pyruvate dehydrogenase activity (Figure 6A), GABAergic synapses (Figure 6B), synaptic signalling and plasticity (Figure 6C) and Wnt signalling (Figure 6D) respectively.

## 4 Discussion

GSK3β has been linked to multiple processes involved in synapse formation, neurotransmission and plasticity [7]. Work from our laboratory has previously shown that modulations of GSK3β abundance or activity alter EtOH behaviours, including consumption [6]. In the present study, we investigated the actions of a highly selective and clinically available GSK3β inhibitor, tideglusib, on two different models of EtOH consumption. Our results show significant and selective effects of tideglusib on EtOH consumption without other persistent associated behavioural, pharmacokinetic or toxicity changes.

Tideglusib has been studied in Phase 2 clinical trials for treatment of Alzheimer's disorder, progressive supranuclear palsy, autism spectrum disorder and myotonic dystrophy 1, and Phase 2/3 trials for congenital myotonic dystrophy are currently underway [13, 14, 15, 16, 17]. The most frequent side effects in these trails were diarrhoea in 13%–18% and an asymptomatic, transient and reversible increase in aminotransferase in 9%–16% of subjects [15, 16, 17]. A clinical trial of tideglusib on myotonic dystrophy also reported mild nasopharyngitis in 31% of patients [15], though this effect was not observed in trials on other disorders. In our studies, we assessed levels of alanine aminotransferase, aspartate aminotransferase and alkaline phosphatase. There was no effect of tideglusib treatment on either aminotransferase; however, we did see a tideglusib‐induced decrease in alkaline phosphatase (Figure S3B–D). Almost the entirety of serum alkaline phosphatase has been found to come from liver or bone, and while the exact function of alkaline phosphatase outside of bone mineralisation is not known, it is most often assessed clinically to help diagnose liver disease in conjunction with the other liver enzymes we assayed. However, in the cases of liver disease, alkaline phosphatase is typically elevated in contrast to the decrease we have observed with tideglusib [28]. Interestingly, previous studies on osteoblast differentiation have also shown GSK3β inhibition to increase alkaline phosphatase activity, hypothesising these effects are mediated through Wnt signalling [29]. Though our liver enzyme analysis was limited to males only, the clinical trials cited above did assess both males and females and did not report sex differences. The findings in all clinical trials are consistent; tideglusib is safe and well tolerated within human populations, a pivotal aspect in furthering the study of tideglusib in the use of AUD.

Here, we have shown tideglusib successfully decreases both EtOH consumption and preference in mice drinking EtOH for prolonged periods within the IEA model. IEA is well documented to result in progressive, increased consumption over time [30]. Such progression is typical of AUD, where initial exposure to alcohol progresses to excessive and compulsive consumption. Our studies in Experiments 2 and 3 suggest that tideglusib may have utility in decreasing drinking behaviours in humans with established problematic EtOH consumption as in AUD. Our first IEA experiment gave male and female mice a single 200 mg/kg dose of tideglusib. This elicited a decrease in drinking behaviours during the binge consumption period in both sexes (Figure 2A–D) but failed to have a significant effect on 24‐h consumption or EtOH preference (Figure 2E–H). This was likely due to a relatively short half‐life of tideglusib in mice, thus explaining the comparatively high doses of tideglusib needed for actions on EtOH consumption in mice compared to humans [15]. While these studies were being conducted, a report determined the half‐life for tideglusib in male Balb/C mice was 5.12 h [31]. We verified this in C57BL/6J mice (4.9 h, data not shown), and therefore, we performed a second IEA experiment where we administered two separate 100 mg/kg doses of tideglusib spread 4 h apart to elicit changes in consumption over a 24‐h period. This dosing paradigm produced significant decreases in daily EtOH consumption and preference (Figure 3), suggesting that pharmacological levels of tideglusib were altering consumption.

Since problematic binge EtOH consumption is frequently seen in humans, we investigated the DID model of binge drinking where mice consume high levels of EtOH within a relatively short time span [32]. Our results showed a dose‐responsive effect of tideglusib on consumption in males and females, with a mild significant increase in potency with males (Figure 4A). Importantly, our DID studies showed a marked decrease in EtOH consumption even after considerable habituation to EtOH drinking and that tideglusib did not have long‐lasting actions on consumption following washout (Figure 4B). This suggests GSK3β inhibition via tideglusib may have utility as an intervention to for blunting recidivism in patient populations recovering from AUD.

Underlying differences in pharmacokinetics, taste perception [33], affective and locomotor behaviours can be associated with differences in EtOH consumption. We therefore conducted control studies to determine possible actions of a high tideglusib dose (200 mg/kg) on these factors. Our studies showed no effect of tideglusib on EtOH pharmacokinetics or taste preference. Tideglusib did produce a transient increase in total distance travelled within the first 5 min of testing (Figure 5F). However, this effect on locomotion dissipated quickly, with no effect on the last 5 min of testing or when the entire 10‐min test period was assessed (Figure S4). Other preclinical studies on GSK3β via genetic knockdown or pharmacological inhibition have shown no effects on locomotion [34, 35] and similarly clinical trials in humans have not reported tideglusib‐induced motor effects in patients [15, 16, 17]. It should be noted in a clinical trial for progressive supranuclear palsy, the patient population displays movement abnormalities pretreatment. However, while tideglusib was ineffective at improving motor function in these subjects, no new or worsening effects on movement were reported [17].

It is well established that there is a high prevalence of comorbidity between AUD and anxiety disorders. One hypothesis as to this relationship states that alcohol produces a reduction in “basal” or withdrawal‐induced anxiety symptoms and that this reduction is a major motivator for chronic drinking behaviour and recidivism [36]. Previous mouse genetic studies which decreased GSK3β levels found contradictory results on basal anxiety‐like responses [35, 37]. In our studies using the light–dark box assay, while we demonstrated the anxiolytic effects of EtOH, there were no effects of tideglusib treatment on anxiety‐like behaviour in the absence or presence of EtOH (Figure 5D,E). The systemic nature of our GSK3β modulations compared to the more targeted approaches used in the studies mentioned above may account for the differences between those reports and the lack of tideglusib effects on anxiety‐like behaviours in our studies [35, 37, 38, 39].

To determine possible downstream mechanisms of tideglusib action on decreasing EtOH consumption, we presented here initial analysis of RNAseq data on animals consuming EtOH chronically with or without tideglusib treatment. Full details of this analysis will be presented elsewhere. Previous work has shown EtOH produces inhibitory phosphorylation of GSK3β at its serine‐9 residue through Akt signalling in the NAc [40], and Akt‐induced GSK3β phosphorylation can promote cell proliferation through activation of mTOR [41]. However, based upon our RNAseq findings, we hypothesised that tideglusib may act on EtOH consumption in an Akt‐independent manner via GSK3β actions on Wnt signalling, particularly through the canonical Wnt‐β‐catenin signalling pathway based upon the transcription factor binding sites overrepresented in our RNAseq analysis of EtOH versus EtOH + tideglusib animals. Importantly, this does not eliminate mTOR signalling as a mechanism for the actions of GSK3B inhibition on EtOH consumption, as Akt‐independent Wnt signalling through GSK3β has also been shown to activate mTOR [41]. Additionally, disturbance of an Akt‐dependent pathway to beta‐catenin signalling is also possible since this has been described as modulating EtOH consumption [40].

We found that the top five TFBS categories overrepresented for genes regulated by tideglusib involved Wnt signalling (Figure 6D). In particular, the two most significant TFBS, transcription factor three (TCF3) and lymphoid enhancer‐binding factor (LEF1), are both binding sites for β‐catenin, a known member of the canonical Wnt signalling pathway along with GSK3β [42]. Interestingly, c‐Myc‐regulated genes were also identified as a top category in our TFBS gene ontology results (Figure 6D), but c‐Myc was not significantly regulated by tideglusib (Table S1). This may be due to the co‐occurrence of c‐Myc or β‐catenin binding sites in a significant number of genes since it is known that these two transcriptional regulators can overlap in their target genes.

When GSK3β is active, it phosphorylates β‐catenin which is subsequently targeted for ubiquitylation. When GSK3β is inhibited, β‐catenin is no longer degraded, allowing it to enter the nucleus and regulate gene expression by binding to the TCF/LEF transcription factor family [42]. Wnt signalling via Wnt‐3a and Wnt‐7a have both been shown to regulate presynaptic plasticity via the canonical Wnt‐β‐catenin signalling pathway by strengthening excitatory synapses [43, 44, 45]. Additionally, both Wnt‐3 and Wnt‐7a have been shown to enhance long‐term potentiation postsynaptically as well [46, 47], and treatment with Wnt‐3a induced transcriptional changes in genes associated with learning and memory and neurotransmitter release [48]. Tideglusib‐induced changes in Wnt actions on both pre‐ and postsynaptic plasticity could account for the gene ontology changes we see in our cellular components and biological processes (Figure 6B,C).

Pyruvate dehydrogenase activity, a main ontology result for molecular functions (Figure 6A, Table S2) from our RNAseq analysis of EtOH versus EtOH + tideglusib, is also linked to both GSK3β and Wnt signalling. Pyruvate dehydrogenase catalyses the oxidation of pyruvate, forming acetyl‐CoA, CO_2_ and NADH (H^+^), and plays a critical role in linking the glycolytic pathway to the oxidative pathway [49]. Pyruvate dehydrogenase has been identified as a substrate of GSK3β [50]. Additionally, pyruvate dehydrogenase kinase (PDK1) has been identified as a Wnt target gene. Blocking Wnt signalling through a dominant negative LEF/TCF isoform downregulates PDK1, an inhibitor of the pyruvate dehydrogenase complex in mitochondria [51]. Interestingly, c‐Myc, another Wnt target gene identified in our TFBS gene ontology results (Figure 6D), was unaffected by the dominant negative LEF/TCF, suggesting that the downregulation of PDK1 was limited to canonical Wnt‐β‐catenin signalling through LEF/TCF3 and not other Wnt signalling pathways [51].

We hypothesise EtOH alters the Wnt‐β‐catenin signalling pathway to regulate neuronal plasticity within mPFC in the IEA progressive EtOH consumption model, and future studies will be aimed at investigating this potential mechanism in other brain regions, such as the NAc or amygdala. GSK3β inhibition may be decreasing consumption by preventing or reversing EtOH‐induced changes to canonical Wnt signalling, resulting in expression changes in genes containing Wnt‐related transcription factor binding sites in our RNAseq analysis of PFC from EtOH versus EtOH + tideglusib animals. Without future mechanistic studies, however, our RNAseq results currently only implicate a beta‐catenin pathway in the actions of tideglusib, and thus, EtOH consumption could involve either Wnt‐ or Akt‐mediated actions, both of which can increase beta‐catenin related transcription regulation.

A limitation of the DEG analysis presented here is the absence of any non–EtOH‐drinking animals to compare effects of tideglusib in animals without an EtOH history. As such, we do not know whether tideglusib alone or the interaction between EtOH and tideglusib is responsible for the changes observed in downstream Wnt signalling genes. Ongoing analysis of further RNAseq studies [20] will clarify this issue. However, as mentioned in the Results section, only a very small number of genes present in our EtOH + tideglusib versus EtOH + vehicle DEG dataset overlapped with genes responding to tideglusib alone in water drinking animals. This suggests that the DEG dataset described here represents largely those EtOH‐responsive genes that are modulated by the GSK3β inhibitor, tideglusib. Additionally, our genomic studies collected brain tissue at 24 h after the last dose of EtOH or tideglusib. Our results could thus represent a combination of chronic EtOH/tideglusib responses in addition to genes regulated by an acute withdrawal from EtOH. We chose this design because we were most interested in identifying the gene expression profile at the time when animals would next have access to EtOH. Lastly, the RNAseq results were limited to males only. The dose of tideglusib used (100 mg/kg) reduced EtOH consumption to similar levels in both sexes in our DID studies and potentially hints at similar transcriptional changes within males and females, though future studies should assess if this is true.

Despite the levels of AUD within the population, only 7.6% of individuals with AUD received any treatment in 2022, and only 2.1% of people with AUD received medication‐assisted treatment [1]. A part of this treatment gap can be attributed to the lack of utilisation and effectiveness of the three current FDA‐approved medications for the treatment of AUD (disulfiram, naltrexone and acamprosate). The data presented here support the use of tideglusib in human clinical studies as an intervention in AUD.

## Author Contributions

Conceived and designed the study: S.G., A.v.d.V., J.T.W., M.F.M. Acquired the data: S.G., A.v.d.V., A.H., D.B., A.M., B.O. Analysed the data: S.G., W.D.R., M.F.M. Wrote the paper: S.G., M.F.M.

## Disclosure

The content is solely the responsibility of the authors and does not necessarily represent official views of the National Institutes of Health.

## Ethics Statement

Rodent animal studies and procedures were approved by the Institutional Animal Care and Use Committee of Virginia Commonwealth University and followed the NIH Guide for the Care and Use of Laboratory Animals.

## Conflicts of Interest

The authors declare no conflicts of interest.

## Supporting information


**Data S1.** Supplemental methods.

**Table S1.** Tideglusib_paper_DEG_results_EtOHTid_vs_EtOHVeh_PFC_supplemental.

**Table S2.** Tideglusib_paper_ontology.

**Figure S1.** Tideglusib treatment has no effect on body weight. (A) There was a main effect of treatment phase (baseline vs. treatment) where mice gained weight over time (*F*_1,35_ = 54.003, *p* < 0.0001) and sex where males were larger than females (*F*_1,35_ = 273.621, *p* < 0.0001) but no effect of tideglusib on body weight nor any interaction. (B) There was a main effect of treatment phase (*F*_1,44_ = 247.912, *p* < 0.0001) but no effect of tideglusib or ethanol on body weight, nor any interaction.
**Figure S2.** Tideglusib is more potent in males than in females. (A) Tideglusib has an ED50 of 64.6 mg/kg (95% CI = 58.9–70.8) and (B) an ED50 of 79.4 mg/kg in females (95% CI = 70.8–93.3). ED50s were calculated using log tideglusib does then transformed back to base mg/kg for reporting purposes. The different ED50s (dotted lines) with nonoverlapping CIs suggest significant differences in potency of tideglusib between sexes.
**Figure S3.** Tideglusib treatment has no effect on ethanol metabolism or aminotransferase levels but increases alkaline phosphatase levels. (A) There was a main effect of time post injection on BEC (*F*_3,18_ = 12.345, *p* < 0.0001) but no effect of tideglusib on ethanol pharmacokinetics as measured by BEC at any timepoint tested (10, 30, 60, 90 min) (*n* = 3–4/group/timepoint). (B, C) Tideglusib has no effect on alanine aminotransferase or aspartate aminotransferase but (D) significantly decreases alkaline phosphatase levels (*F*_1,12_ = 20.018, *p* = 0.0008) (*n* = 3–4/group). Tukey post hoc analysis revealed significant differences between H2O‐Veh versus H2O‐TID and EtOH‐VEH versus EtOH‐TID (*p < 0.05). There was no effect of ethanol on any enzyme measured nor an interaction between ethanol and tideglusib.
**Figure S4.** Tideglusib treatment has no effect on ethanol‐induced anxiolysis or total locomotion over 10‐min LDB testing. (A) i.p. ethanol significantly decreases anxiety‐like behaviour in the light–dark box as measured by percent time in light (*F*_1,50_ = 7.204, ^##^ *p* < 0.01) and (B) percent distance in light (*F*_1,50_ = 10.337, ^##^ *p* < 0.01) with no effect of sex or tideglusib and no interactions (*n* = 4–8/group). No comparisons reached significance in post hoc testing. (C) There is no effect of ethanol, tideglusib or sex on total distance travelled when measured over the entire 10‐min testing interval.

## Data Availability

The data that support the findings of this study are available from the corresponding author upon reasonable request.
