# Supplementary material for: A Selective GSK3β Inhibitor, Tideglusib, Decreases Intermittent Access and Binge Ethanol Self‐Administration in C57BL/6J Mice
Source: Addict Biol. 2025 May 19;30(5):e70044. doi: 10.1111/adb.70044 (PMC12089657; doi:10.1111/adb.70044)
Supplement: Supplementary file 1 — Data S1. Supplemental methods. [file ADB-30-e70044-s004.docx]

**Supplemental Methods**

RNA isolation and sequencing

Mice were euthanized 24 hours after their last ethanol access via cervical dislocation and decapitation. PFC was collected, flash-frozen in liquid nitrogen, and stored at -80° C. RNA was extracted and purified using RNeasy mRNA kits (Qiagen, Germantown, MD). Quality of isolated RNA was assessed using both a NanoDrop assay (Thermo Fisher Scientific, Waltham, MA) and BioAnalyzer 2100 (Agilent Technologies, Santa Clara, CA). Isolated RNA samples were then shipped on dry ice for poly-A selected library construction and sequencing on an Illumina NextSeq 2000 platform with P3 flow cell (Illumina, Inc. San Diego, California) in a strand-specific 100bp paired-end sequencing reaction at the VCU Genomics Core.

Default read quality control, duplicate sequence identification, adapter sequence trimming, and G-C content settings were used for fastP processing (v0.23.2). Read strandedness was determined by default parameters in the how_are_we_stranded_here Python library (v1.0.1). Reads were aligned to GRCm39 genome by STAR (v2.7.10b). Sorting of BAM files and downstream indexing was performed with Samtools (v1.5.1). The featureCount program in the Subread package (v2.0.1) was used to quantify aligned reads. Samples were screened for outliers by principal component analysis and visualization of variance stabilized-transformed reads.
