# Supplementary material for: A Selective GSK3β Inhibitor, Tideglusib, Decreases Intermittent Access and Binge Ethanol Self‐Administration in C57BL/6J Mice
Source: Addict Biol. 2025 May 19;30(5):e70044. doi: 10.1111/adb.70044 (PMC12089657; doi:10.1111/adb.70044)
Supplement: Supplementary file 4 — Figure S1. Tideglusib treatment has no effect on body weight. (A) There was a main effect of treatment phase (baseline vs. treatment) where mice gained weight over time (F 1,35 = 54.003, p < 0.0001) and sex where males were larger than females (F 1,35 = 273.621, p < 0.0001) but no effect of tideglusib on body weight nor any interaction. (B) There was a main effect of treatment phase (F 1,44 = 247.912, p < 0.0001) but no effect of tideglusib or ethanol on body weight, nor any interaction. Figure S2. Tideglusib is more potent in males than in females. (A) Tideglusib has an ED50 of 64.6 mg/kg (95% CI = 58.9–70.8) and (B) an ED50 of 79.4 mg/kg in females (95% CI = 70.8–93.3). ED50s were calculated using log tideglusib does then transformed back to base mg/kg for reporting purposes. The different ED50s (dotted lines) with nonoverlapping CIs suggest significant differences in potency of tideglusib between sexes. Figure S3. Tideglusib treatment has no effect on ethanol metabolism or aminotransferase levels but increases alkaline phosphatase levels. (A) There was a main effect of time post injection on BEC (F 3,18 = 12.345, p < 0.0001) but no effect of tideglusib on ethanol pharmacokinetics as measured by BEC at any timepoint tested (10, 30, 60, 90 min) (n = 3–4/group/timepoint). (B, C) Tideglusib has no effect on alanine aminotransferase or aspartate aminotransferase but (D) significantly decreases alkaline phosphatase levels (F 1,12 = 20.018, p = 0.0008) (n = 3–4/group). Tukey post hoc analysis revealed significant differences between H2O‐Veh versus H2O‐TID and EtOH‐VEH versus EtOH‐TID (*p < 0.05). There was no effect of ethanol on any enzyme measured nor an interaction between ethanol and tideglusib. Figure S4. Tideglusib treatment has no effect on ethanol‐induced anxiolysis or total locomotion over 10‐min LDB testing. (A) i.p. ethanol significantly decreases anxiety‐like behaviour in the light–dark box as measured by percent time in light (F 1,50 = 7.204, ## p < 0.01) a [file ADB-30-e70044-s003.docx]

**SUPPLEMENTAL FIGURES**

**
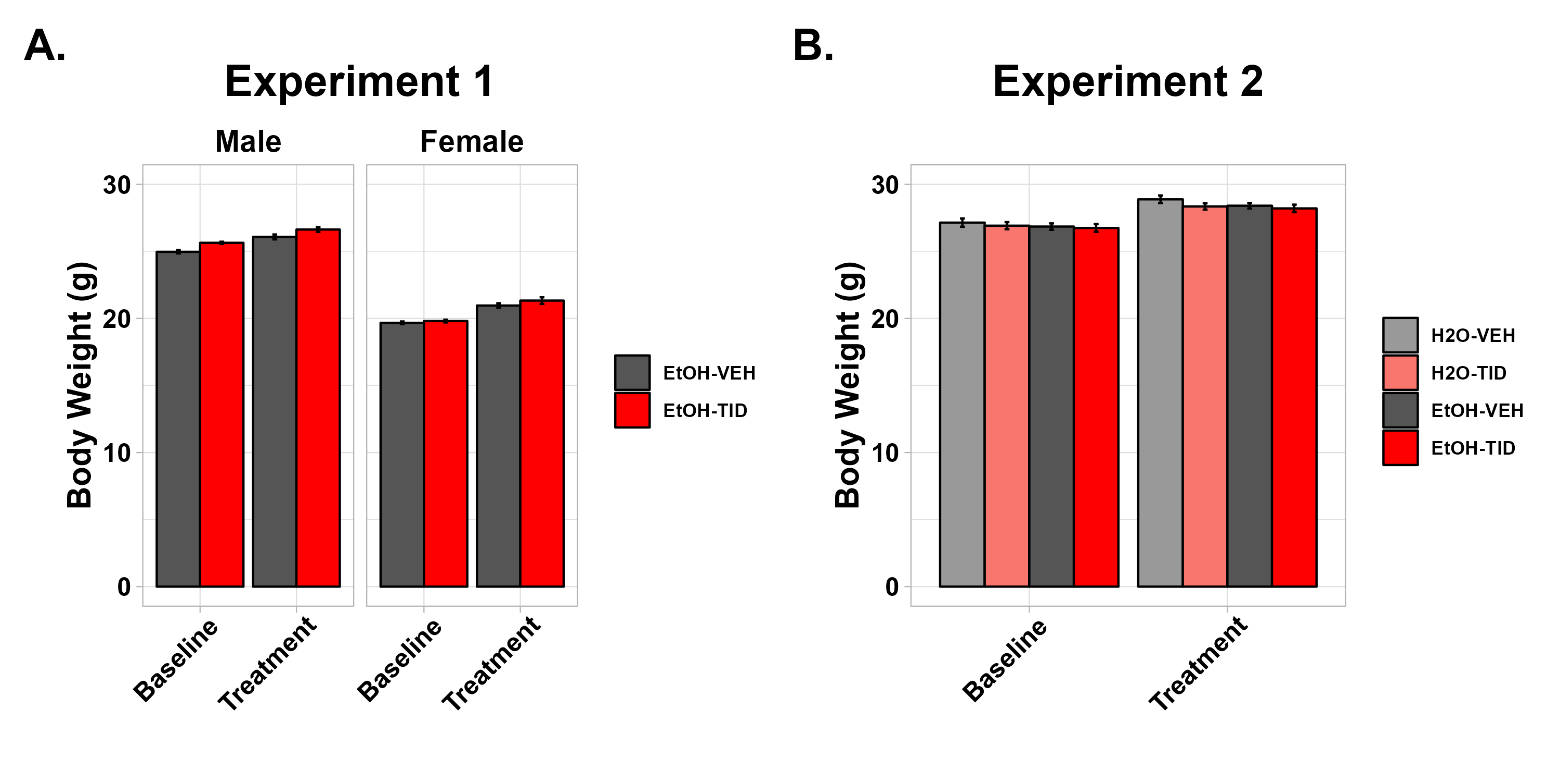
 Supplemental Figure 1.** Tideglusib treatment has no effect on body weight. **A** There was a main effect of treatment phase (baseline versus treatment) where mice gained weight over time (F_1,35_=54.003, p<0.0001) and sex where males were larger than females (F_1,35_=273.621, p<0.0001) but no effect of tideglusib on body weight nor any interaction. **B** There was a main effect of treatment phase (F_1,44_=247.912, p<0.0001) but no effect of tideglusib or ethanol on body weight, nor any interaction.


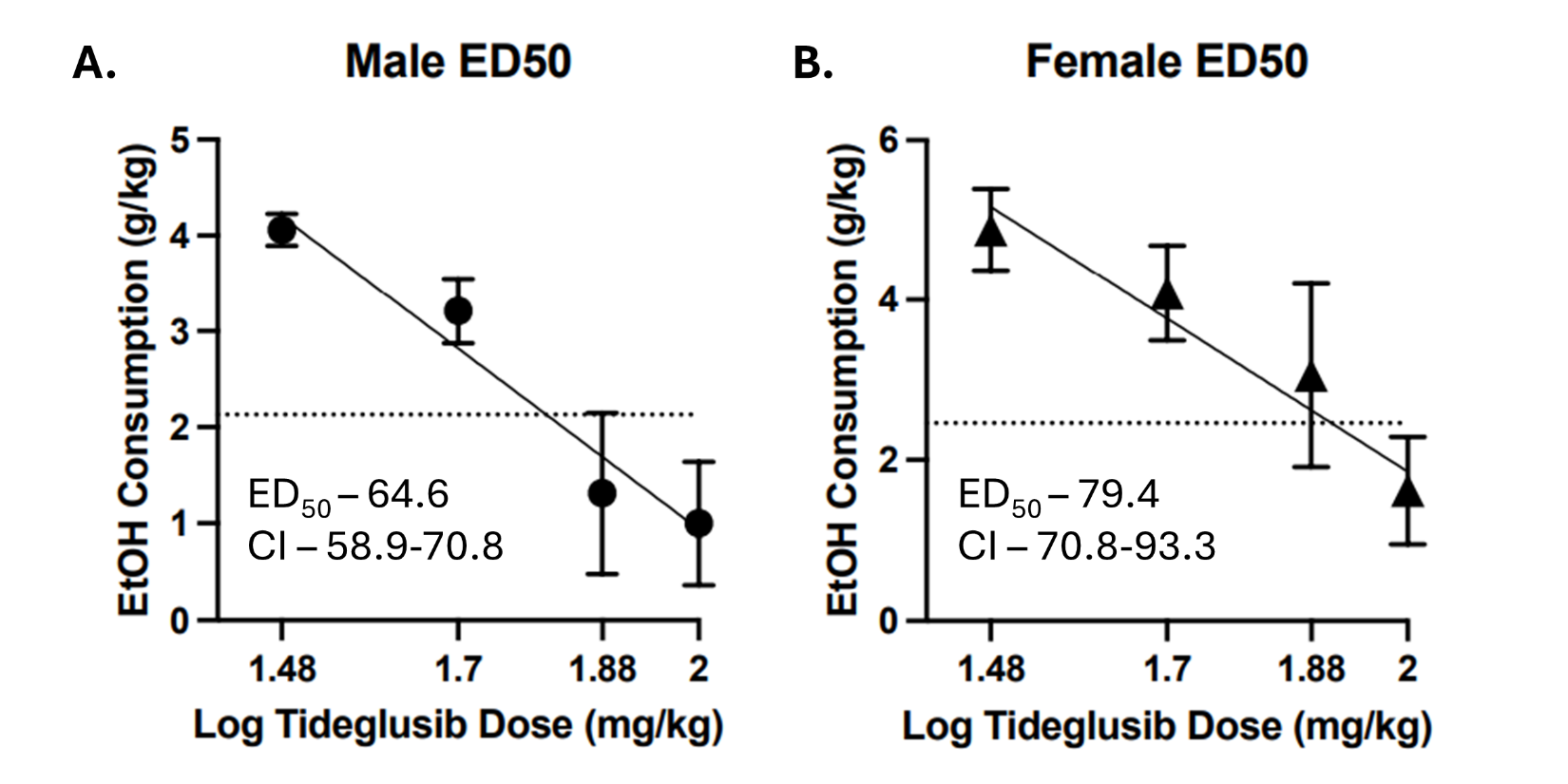


**Supplemental Figure 2.** Tideglusib is more potent in males than females. **A** tideglusib has an ED50 of 64.6mg/kg (95% CI=58.9-70.8) and **B** an ED50 of 79.4mg/kg in females (95% CI=70.8-93.3). ED50s were calculated using log tideglusib does then transformed back to base mg/kg for reporting purposes. The different ED50s (doted lines) with non-overlapping CIs suggests significant differences in potency of tideglusib between sexes.

**
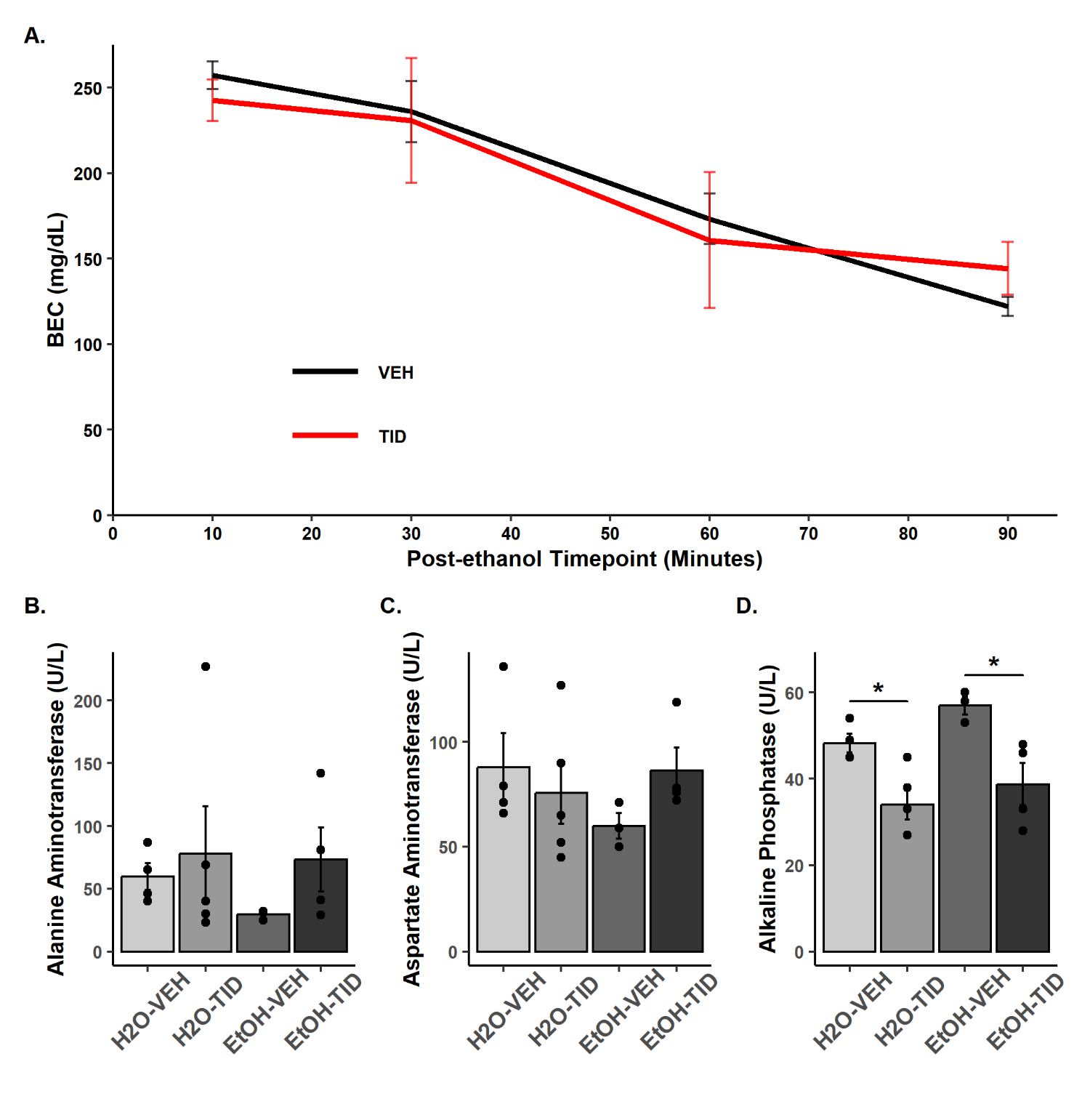
Supplemental Figure 3.** Tideglusib-treatment has no effect on ethanol metabolism or aminotransferase levels but increases alkaline phosphatase levels. **A** There was a main effect of time post injection on BEC (F_3,18_= 12.345, p<0.0001) but no effect of tideglusib on ethanol pharmacokinetics as measured by BEC at any timepoint tested (10, 30, 60, 90min) (n=3-4/group/timepoint). **B,C** Tideglusib has no effect on alanine aminotransferase or aspartate aminotransferase but **D** significantly decreases alkaline phosphatase levels (F_1,12_=20.018, p=0.0008) (n=3-4/group). Tukey posthoc analysis revealed significant differences between H2O-Veh vs H2O-TID and EtOH-VEH vs EtOH-TID (*p<0.05). There was no effect of ethanol on any enzyme measured nor an interaction between ethanol and tideglusib.

**Supplemental Figure 4.** Tideglusib-treatment has no effect on ethanol-induced anxiolysis or total locomotion over 10-min LDB testing. **A** i.p. ethanol significantly decreases anxiety-like behavior in the light-dark box as measured by percent time in light (F_1,50_=7.204, ##p<0.01) and **B** percent distance in light (F_1,50_=10.337, ##p<0.01) with no effect of sex or tideglusib and no interactions (n=4-8/group). No comparisons reached significance in post hoc testing. **C** There is no effect of ethanol, tideglusib, or sex on total distance traveled when measured over the entire 10-min testing interval.**
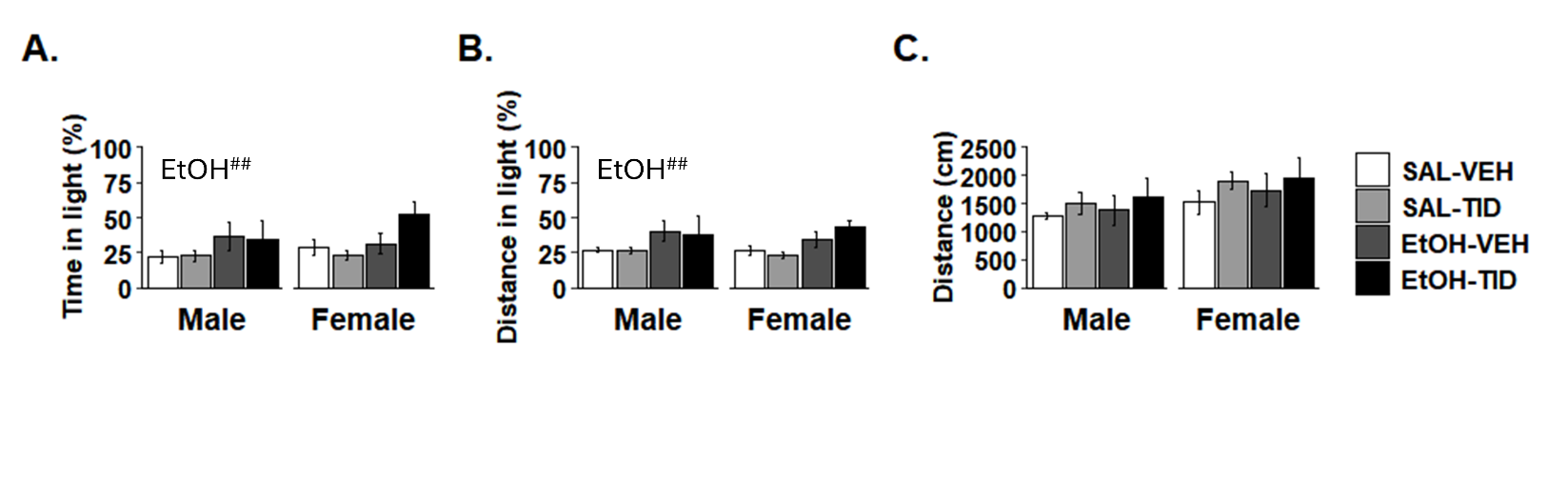
**
